# Overview of technology developments in probiotic field

**DOI:** 10.3402/mehd.v23i0.18561

**Published:** 2012-06-18

**Authors:** Catherine Stanton, Gerald F. Fitzgerald, R. Paul Ross

**Affiliations:** 1 TEAGASC, Moorepark Food Research Centre, Fermoy, Ireland; 2 Alimentary Pharmabiotic Centre, Cork, Ireland; 3 Microbiology Department, University College Cork, Cork, Ireland

## Abstract

Probiotics are ‘live microorganisms which, when administrated in adequate amounts, confer a health benefit on the host’ (FAO/WHO, 2001). This requirement, i.e. that the probiotic bacteria must be in viable form at the time of consumption, poses a number of technical challenges from food processing perspectives. Environmental stresses encountered during food processing include acid exposure during food fermentations, extremes in temperatures encountered during drying processes, in addition to oxidative, osmotic, and food matrix stresses. Furthermore, the ingested bacteria must remain viable during gastric transit, to reach the site of action in viable form to exert the probiotic effects. This imposes further stresses, as the gastrointestinal tract is naturally designed to impede the passage of microorganisms with low pH encountered in the stomach and the detergent-like properties of bile encountered in the duodenum. A number of approaches have been investigated in order to minimise the damage caused by exposure to such stresses experienced by probiotics during food processing and gastric transit. Approaches for protection of probiotic viability during food processing and shelf life include manipulation of bacterial cell physiology, application of prelethal stress to the cultures during cell preparation, selection of appropriate drying conditions, and optimisation of reconstitution conditions after drying. Furthermore, probiotic viability losses can be minimised by selection of appropriate food carriers for their delivery to the intestine. In this respect, the composition and physical nature of the food matrix can have profound effects on the stability of live probiotics during gastric transit. Encapsulation of probiotics is another approach to positively affect viability of probiotics in some matrices. Furthermore, it is important to understand the mechanisms underlying bacterial survival in hostile environments in order to develop efficacious functional foods delivering the benefits associated with the probiotics within.

